# “I think we still do too little”: measures to prevent violence and aggression in German emergency departments – a qualitative study

**DOI:** 10.1186/s12913-023-09044-z

**Published:** 2023-01-30

**Authors:** Sonja Reißmann, Tanja Wirth, Vanessa Beringer, David A. Groneberg, Albert Nienhaus, Volker Harth, Stefanie Mache

**Affiliations:** 1 Institute for Occupational and Maritime Medicine (ZfAM), University Medical Center Hamburg-Eppendorf (UKE), 20459 Hamburg, Germany; 2 Institute of Occupational Medicine, Social Medicine and Environmental Medicine, Goethe University Frankfurt, 60590 Frankfurt, Germany; 3 Department of Occupational Medicine, Hazardous Substances and Public Health, Institution for Statutory Accident Insurance and Prevention in the Health and Welfare Services (BGW), 22089 Hamburg, Germany; 4 Institute for Health Services Research in Dermatology and Nursing (IVDP), Competence Center for Epidemiology and Health Services Research for Healthcare Professionals (CVcare), University Medical Center Hamburg-Eppendorf (UKE), 20246 Hamburg, Germany

**Keywords:** Violence, Prevention, Emergency department, Occupational health, Occupational safety, Qualitative research

## Abstract

**Background:**

Healthcare workers employed in emergency departments (EDs) are particularly affected by physical and verbal violence. Violent assaults can be committed by both patients and their attendants. Research on interventions for violence prevention is limited and previous studies report that ED employees feel unprepared for violent incidents. Thus, the current study aims to explore ED staff’s perceptions regarding available prevention measures, their effectiveness, barriers, and further needs in terms of violence prevention.

**Methods:**

In accordance with the qualitative study design, 27 semi-structured interviews were conducted via telephone with doctors and nurses working in direct contact with patients in German EDs. Main subjects were advantages and disadvantages of currently available measures, barriers regarding their implementation, their perceived effectiveness, as well as further needs concerning violence prevention. The transcribed interviews were analysed according to Mayring’s qualitative content analysis.

**Results:**

Participants described environmental (e.g., alarm systems), organisational (e.g., security service), and individual-focused measures (staff training, verbal de-escalation). Measures perceived as effective were, for instance, communication and security service. Both demands and barriers were often related to financial constraints, e.g., staff shortage led to higher workloads and less time to consider violence prevention. In most cases, guidelines or standard operating procedures (SOPs) regarding violence prevention were missing, unknown, or not perceived as helpful in their current form. Furthermore, screening tools were not applied in any of the EDs.

**Conclusions:**

The workload in EDs needs to be decreased in order to enable violence prevention, e.g., by reducing patient inflow or by increasing personnel. In addition, violence prevention guidelines tailored to the requirements of the respective ED need to be developed. Hospitals should supply ED staff with such guidelines, e.g., in the form of SOPs, but more importantly, prevention measures have to be practiced and communicated. Furthermore, there is a need for research on the implementation of screening tools for violent behaviour, so that the focus would shift from managing violence to preventing violence.

## Background

Healthcare professionals experience workplace violence, and those who work in emergency departments (EDs) are found to be particularly affected [1, 2]. According to the International Labour Organization (ILO) “the term ‘violence and harassment’ in the world of work refers to a range of unacceptable behaviours and practices, or threats thereof, whether a single occurrence or repeated, that aim at, result in, or are likely to result in physical, psychological, sexual or economic harm, and includes gender-based violence and harassment” [3]. In their systematic review and meta-analysis Liu et al. [2] report on workplace violence in several healthcare settings across the world. For ED settings, the 12-month prevalence amounts to 79.4% (95% confidence interval [CI], 75.2–83.6) for any kind of violence, while physical and non-physical violence (i.e., verbal abuse, threats, or sexual harassment) account for 31.0% (95% CI, 26.0–36.0) and 62.3% (95% CI, 53.7–70.8), respectively [2]. Perpetrators of violence include either the patients themselves or their attendants [4].

Consequences of workplace violence for healthcare staff are physical and emotional injuries [5, 6]. Moreover, exposure to violence has an impact on the professional quality of life, as Copeland and Henry [7] describe increased levels of burnout and secondary traumatic stress, as well as decreased levels of compassion satisfaction, e.g., for exposure to non-physical violence such as threats. Apart from this, there are consequences for employers (e.g., missed workdays [8]) and patients (e.g., reduced quality of care [6]) as well.

Several guidelines on violence prevention are available, like the joint programme on workplace violence in the healthcare sector [9], which summarises possible interventions. These interventions target violence prevention on various levels, i.e., preconditions (e.g., workplace culture), after-the-event interventions (e.g., reporting), environmental measures (e.g., alarm systems), organisational measures (e.g., work practices), and individual-focused measures (e.g., training) [9].

Recently, a systematic review on interventions for violence prevention in EDs was conducted by Wirth et al. [10]. Out of 15 included studies, 11 report on individual-focused behavioural interventions such as de-escalation skills, while only 4 studies provide results on organisational or environmental measures in addition to educational components [10]. Considering the small amount of available literature as well as methodological weaknesses of included studies, Wirth et al. [10] conclude that more research is needed. Already in 2010, Anderson et al. [11] called for a shift from defining the phenomenon of violence in EDs to investigating potential interventions. In addition, several studies from Germany show that only few staff members feel well prepared [12], while the majority of ED staff feel unprepared for violent situations [13, 14]. Hence, the aim of the current study is to explore how employees perceive measures to prevent violence and aggression in German EDs, with a focus on environmental, organisational, and individual-focused measures. This leads to the following research questions:

1. Which measures to prevent violence and aggression caused by patients and their attendants are already known by medical and nursing ED employees in German EDs?
2. Which violence prevention measures are perceived as effective by German medical and nursing ED employees?
3. Which barriers and enablers regarding the implementation of violence prevention measures are expressed by medical and nursing ED employees in Germany?
4. What do German medical and nursing ED employees identify as further needs regarding violence prevention?

## Methods

### Study design

A qualitative study design was chosen to explore the participants’ perspectives on different violence prevention measures. The qualitative approach added depth to the findings, by learning about participants’ perceptions regarding the advantages and disadvantages of available preventive measures or further needs. Moreover, due to the qualitative study design it was possible to gain an insight into the influence of practitioners’ personal beliefs or their behaviour on violence prevention. This study was guided by the Consolidated Criteria for Reporting Qualitative Research (COREQ) [15].

### Participants and recruitment

Employees and supervisors working as nurses or doctors in German EDs were recruited for the study. Further eligibility criteria were defined as having work experience of at least 6 months in the current ED and working in direct contact with patients and their attendants. Administrative ED-staff was excluded due to the difference in work tasks and responsibilities. Recruitment of participants was supported by members of the working group against violence of the German Society for Interdisciplinary Emergency and Acute Medicine (Deutsche Gesellschaft für Interdisziplinäre Notfall- und Akutmedizin, DGINA), who were also part of the research team. At first, a purposeful sampling technique was applied to identify information rich cases. Participants were then asked to pass on the interview invitation to their colleagues (snowball sampling). In addition, EDs from all emergency care levels according to the Federal Joint Committee (Gemeinsamer Bundesausschuss, G-BA) [16] were considered, to take account of structural preconditions (e.g., size of the ED), which might have an influence on the availability of preventive measures. The emergency care levels according to the G-BA are assigned to hospitals that have a central emergency department. This classification divides EDs into basic, enhanced, or comprehensive care levels and is, for instance, based on available medical equipment and specialties, as well as amount and qualification of staff [16].

### Data collection

A semi-structured interview guideline was prepared beforehand, containing questions about current violence prevention measures, their perceived effectiveness and further needs regarding violence prevention (c.f. Table 1).

**Table 1:** General topics addressed in the semi-structured interview guideline

Section	General subject^**a**^	Specific topic
**1**	Data on demographics and occupation	- Age, gender, working hours, profession, work experience
**2**	Prevention measures known by participants	- Measures to identify aggressive patients and to deal with them on the environmental, organisational, and individual-focused level - Support in dealing with aggressive patients
**3**	Implementation of measures	- Perceived effectiveness of measures - Perceived barriers regarding implementation
**4**	Further needs	- Demands to prevent or reduce violent incidents on the environmental, organisational, and individual-focused level

^a^The interview guideline comprised further topics, which will be presented elsewhere, e.g., post-incident measures, reporting of incidents, as well as supervisors’ and hospital leaders’ role in violence prevention

The guideline was discussed with all members of the research team as well as verified regarding comprehensibility of questions and flow of conversation during a test interview with an emergency care doctor.

Altogether 27 interviews were conducted between June and September 2021. The interviews were conducted by one female researcher (SR) with a background in public health, who worked in the research area of occupational mental health during the study period. Due to the COVID-19 pandemic, the interviews were conducted via telephone, allowing for the interviewees to choose the right time and a safe place for the interview. Some interviewees chose to participate during working hours, while others preferred to participate from home. No interviewee dropped out or refused to take part in the study. The interviews took 55 minutes on average, varying between 23 to 101 minutes. All interviews were audio recorded after the participants gave their informed consent. The data collection ended once the theoretical saturation was reached. No repeat interviews were carried out and neither the transcripts nor the findings were returned to the participants for comments or corrections.

### Data analysis

The interviews were transcribed according to the rules provided by Kuckartz [17] and anonymised during this process. Afterwards, qualitative content analysis according to Mayring [18] was applied. More specifically, the approach of content structuring was used, i.e. main categories were deductively derived based on the interview guideline, and the material was summarised for each content dimension; this process was complemented by inductive formation of subcategories [19]. The software MAXQDA was used to encode and manage the data (version 12, VERBI GmbH, Berlin, Germany). One interview was coded by two researchers (TW and SR) and dissimilar codings were discussed until consensus was reached. All interviews were then coded by one researcher (SR), whereas ambiguous codings were discussed in the research team. Following Mayring’s [18] approach, all segments were then paraphrased, generalised and reduced to further structure the content. All quotes underpinning the results were translated from German to English by the research team.

## Results

### Characteristics of the study population

The participants (*N* = 27) worked in 19 different EDs located in 8 federal states across Germany. Fifteen interviewees were female compared to 12 male participants, while there was an equal distribution between doctors (*n* = 13) and nurses (*n* = 14). Demographic data and participant characteristics can be found in Table 2.

**Table 2:** Participant characteristics (*N* = 27)

Characteristics	n	%^**a**^
Gender		
Male	12	44
Female	15	56
Age (in years)		
20–29	3	11
30–39	8	30
40–49	6	22
50–59	10	37
Occupation in the ED		
Doctor	13	48
Nurse	14	52
Medical or nursing leadership position in the ED		
Yes	16	59
No	11	41
Work experience in the current ED (in years)		
< 1	2	7
1–5	14	52
6–10	6	22
11–15	2	7
> 15	3	11
Working hours in the ED		
Full-time (≥35 h/week)	24	89
Part-time (< 35 h/week)	3	11
Working in shifts in the ED		
Yes (shifts during nights and/or weekends)	16	59
No (work only on weekdays during the day)	11	41
Emergency care level^b^ of the current ED		
1 (basic care level)	8^c^	29
2 (enhanced care level)	7^c^	25
3 (comprehensive care level)	13^c^	46
Sponsor of institution		
Commercial sponsor (profit-oriented)	4	15
Public sponsor	12	44
Independent sponsor (non-profit, charity, church)	11	41

^a^May not add up to 100% due to rounded numbers

^b^According to the Federal Joint Committee (Gemeinsamer Bundesausschuss, G-BA) [16]

^c^Adds up to *N* = 28 EDs as one interviewee was responsible for two ED-sites with different care levels belonging to the same hospital

### Violence prevention measures

The measures addressed by interviewees were structured into environmental measures (divided into technical and architectural), organisational measures, and individual-focused measures (divided into employees as well as patients and attendants) (cf. Table 3). The following sections provide a summary of participants’ experiences with these violence prevention measures in terms of perceived effectiveness, barriers regarding their implementation, and further demands.

**Table 3:** Violence prevention measures addressed by participants

Environmental measures		Organisational measures	Individual-focused measures	
Technical	Architectural		Employees	Patients and attendants
**Alarm systems** - Permanently installed or carried devices - Loud or silent alarm	**General structure of the ED** - Patient flow - Open and calming atmosphere - Secured equipment - Means of escape	**Staff organisation** - Exchanging the responsible colleague - Supporting colleagues during violent incidents - Attending potentially aggressive patients	**Training regarding violence prevention** - Education on causes of violence, early warning signs, verbal de-escalation, or self-defence	**Communication** - Patient information (e.g., explaining waiting periods) - Means of information and entertainment (e.g., video screens, posters, or patient information systems)
**Locking systems** - Restricted access to (the core area of) EDs	**Registration and waiting areas** - Registration area: welcoming design while considering staff safety - Waiting area: either outside of the ED or inside to make the workload visible; providing water and snacks	**Security service** - Intervening verbally and physically - Quick availability	**Staff care** - Support from supervisors - Promoting stress resistance	**Access restrictions** - Expelling aggressive patients and attendants from the ED - Restricting access for attendants
**Records of violent incidents** - Free-text entries, protocols, or attributes assigned in patient files		**Police** - Last resort, mostly called in cases of physical violence		
**Camera surveillance** - For crucial areas inside and outside of the ED	**Treatment rooms** - Single rooms, especially for individuals with substance misuse or psychiatric patients	**Guidelines and standard operating procedures (SOPs)** - Written instructions regarding violence prevention		**Physical restraint** - As a last resort to prevent aggressive patients from hurting themselves or others
		**Screening tools** - Assigning higher urgency to anxious or aggressive patients		
		**Structural conditions** - Specialised treatment centres for individuals with substance misuse - Outpatient clinics		

#### Environmental measures – technical

##### Alarm systems

The most mentioned technical preventive measures were alarm systems. These existed in the form of permanently installed emergency buttons and bell systems, or wireless telephone and pager systems provided with emergency buttons, as well as acoustical pocket alarm devices. Alarms were either loud to alert colleagues in the direct surrounding or silent to inform specific people about the violent incident (e.g., security staff or police), even without the aggressor noticing. Alarm systems perceived as effective included pager systems and telephones that worked as an intercom or initiated automated notifications including the location after pressing the emergency button.

“Well, we all have telephones with us and there is an, so to speak, emergency button, where you can speak freely, so that you don’t need to have the telephone in your hand. Instead, when I press it (…), I think some kind of security service gets connected, which can follow the conversation via speaker.” [#5, female ED nurse]

Furthermore, it was described that carrying these devices provided a sense of security and thus facilitate confidence in demeanour. Several disadvantages of alarm-systems were discussed. Permanently installed emergency buttons or bell systems might be out of reach, while using silent alarms with an intercom device required the person on the other side to be very cautious, since the aggressor might be triggered on noticing that an alarm was initiated. Wireless pagers or telephones with an emergency feature needed to be available in adequate quantity, which was expensive. Demands regarding technical measures mostly pertained to further expansion of alarm systems, e.g., permanently installed alarm buttons to improve reachability. Furthermore, advanced pager and telephone systems were in demand, just as more simple portable alarm devices to equip each employee with them.

##### Locking systems

Locking systems were described as an effective tool to keep violent incidents outside of the ED. In such cases, patients and their attendants had to be granted access to the ED through a doorbell system, otherwise access was only possible with codes, key cards, or transponders for authorised persons. Especially at night, these locking systems helped employees to feel safe.

“(…) our entrances are secured, so all entrances are locked at night. So then you can only get in with a key from the building or via a numeric code at the entrance of the emergency department, which only the ambulance service, the police and us internal people have. (…) I was at a different hospital before and there all doors were open at night. (…) There I often felt much more insecure on the ward, I have to say.” [#18, female ED nurse]

Other participants reported that only the core area enclosing the treatment rooms was secured from unauthorised access by locking systems and thus, separated from the registration area and the waiting room.

##### Records of violent incidents

Records of previous violent incidents or respective house bans imposed on patients or their attendants were described as a helpful early warning.

“(…) our computer system that we use for the treatment, there is a comments section. It is often filled out directly during the first contact, if there are any incidents that is also saved. Everyone can also access it, who is currently involved in the treatment.”[#18, female ED nurse]

Negative statements regarding these records were related to their visibility and thus effectiveness in warning colleagues before contact with the patient. Hence, highlighted warnings equal to those generally indicating crucial medical conditions were described as more helpful compared to entries hidden in the patient file. Some participants reported that entries were more often used in cases of physical compared to verbal violence and others rarely or never documented violent incidents as warnings for their colleagues.

##### Camera surveillance

Cameras were either discussed for implementation or already installed to monitor critical areas inside (e.g., in waiting areas or treatment rooms to monitor people with substance misuse without being present) or in front of the ED (e.g., in entrance areas or in arrival areas for ambulances). Camera surveillance was also considered as an option for deterrence if signposted. In two cases, the video recordings could be supervised from the nurses’ room, allowing to watch colleagues who were treating a potentially aggressive patient. Critical statements regarding camera surveillance elaborated interference with data protection of both patients and staff members.

“I would like enhanced video surveillance. In principle, we are technically prepared for it, but (…) there are, if you set up cameras, conflicts of interest between staff safety and personal privacy, which the staff council evaluates.” [#6, male ED physician]

#### Environmental measures – architectural

##### General structure of the ED

Some general construction measures were described to facilitate violence prevention. Different patient groups (arriving with the ambulance or by foot) were supposed to enter separately to promote a calmer atmosphere and shorter waiting periods. An open and welcoming design of EDs was addressed as well, such as pleasant and calming wall colours or good lighting conditions. Furthermore, participants described how they tried to prevent equipment from being used as weapons: for instance, seats were fixed to the walls, glass bottles were replaced by plastic bottles, and treatment utensils or employees’ workstations were kept on carts outside the room. However, participants reported that while working on a fixed desk, they had to turn their back to the patient, or that the desk was positioned opposite to the door, thereby blocking the means of escape. Thus, they found it helpful if rooms or counters had a second door to escape. Furthermore, glass encasement of workstations was demanded, allowing for a safe space with the possibility to keep an eye on the surrounding. Some EDs had one specific escape room, in other EDs all rooms were lockable and could be used to escape, but also to lock in highly aggressive patients until the police arrived. Moreover, short distances within the ED were described as helpful to prevent isolated work in secluded areas of the ED. However, there were statements on violence prevention not being a priority when constructing EDs and especially old structural conditions limited the possibilities to implement architectural preventive measures.

“Then we took various other measures, we banned water bottles and we looked at the spatial orientation of the desks in the rooms (…), but that is all very difficult and not 100 percent feasible, (…) because simply the structural conditions are sometimes as they are.” [#27, male ED physician]

In addition, one participant addressed the need for a separate room for staff so that employees could have a break in a calm atmosphere to reduce their stress level.

##### Registration and waiting areas

Well-designed reception areas were described as open, so that patients felt welcome and had a contact person available. Protection of employees in registration areas was described as an important concern, e.g., by installing means of escape and shatterproof glass around counters.

“(…) our base for example, it is a closed room, but where the patients (…) also step up to the counter. And if they (…) come behind the counter – which is no problem – then you have one sole chance, to jump out of the window (…). There is no second exit at the moment, which is impractical.” [#5, female ED nurse]

A central registration and triage area further had the benefit of having all areas within range of vision in order to notice colleagues needing support. Two different concepts regarding waiting areas were described as beneficial. First, an open design (e.g., glass encased) so that waiting people can see the busy ED routines and understand why they have to wait, or second, keeping waiting rooms outside the core area of the ED to prevent impatient or aggressive patients from entering. A one-way-system with several waiting areas was suggested for implementation, meaning that patients would always move forward and never come back to the same waiting room, preventing them from getting frustrated over feeling stuck in slow ED processes.

##### Treatment rooms

Regarding the treatment rooms, an effective violence prevention measure was the possibility to separate potentially aggressive patients and put them in single rooms with minimum facilities and lockable doors. These could be used for patients with psychiatric complaints or intoxication to recover in a low-stimulus environment, while at the same time protecting staff and other patients.

“We can accommodate the people in a low-stimulus environment, we can put them in a single room. Like this they cannot get close to another patient that fast, start a fight with him. We have the possibility, (…), that we can let him sleep calmly and still supervise him with monitors in every room, we can sober them up properly and these are the main patients I am concerned with. And this sobering up in a stable, medical setting, but also in a calm, shielded atmosphere, that is what often helps (…).” [#3, male ED physician]

An important concern were insufficient treatment capacities for the high number of patients. Thus, fast track rooms were planned in one ED to efficiently handle less ill patients so that they do not have to wait. The importance of day light in treatment rooms was emphasised as well, so that patients did not become disorientated while waiting.

#### Organisational measures

##### Staff organisation

Organisational measures described by participants pertained to the organisation of staff and their tasks regarding violence prevention. In some EDs, there was one specific employee responsible for violence prevention who, for instance, organised de-escalation training, reported incidents, and passed on demands expressed by staff members to supervisors. Participants further described that they treated potentially aggressive patients in pairs, left doors of treatment rooms open, or interchanged the responsible personal with a more experienced colleague, a colleague trained in de-escalation, or the supervisor.

“So it is actually like that, especially when it comes to communication, then the nursing colleagues directly go to the doctors’ level or to the nurses’ supervisor level (…). If someone gets abusive, then it is being said: ‘I am leaving and I am coming back with my supervisor’ or ‘I am leaving, I am coming back with the doctor, the doctor will discuss this with you now’.” [#26, male ED physician]

Likewise, female participants shared that they called their male colleagues for help, e.g., in cases of sexual harassment. Calling the quality or complaint management to get a third perspective from someone who could mediate was reported as well. Preliminary verbal warnings about aggressive patients were also common, either from colleagues, or from paramedics and police officers during admission to the hospital. In case of a violent assault, immediate help was provided by all available ED colleagues and the manpower was further increased by calling staff from other wards, especially psychiatric wards. It was reported that during the daytime there was enough staff around to help, but participants also told that this was different at night, especially in smaller EDs. Regarding staff rostering, the participants described several factors. For instance, the registration desk should be staffed with employees trained in de-escalation to calm down aggressive patients and recognise early warning signs for aggressive behaviour right from the beginning. In addition, it was described as beneficial if experienced and trained colleagues were available in every shift, as well as a mix of male and female colleagues. In general, fast ED-processes were perceived as helpful to prevent aggression caused by long waiting times, e.g., fast registration and triage for patients to be informed about their medical situation. However, the high number of patients exceeding ED capacities was addressed, causing long waiting times, leading to aggression, and also impeding the application of preventive measures. Hence, almost all participants addressed the need for more staff in EDs. While a lack of nursing staff was highlighted, an additional shortage of doctors was recognised to cause long waiting times.

“Nursing staff as a whole in the emergency department is too little, yes. The idea in Germany that one is supposed to cover the most severe scenarios with a minimal skeleton crew, that is downright bizarre. And then it must be clear to you that an aggressive patient in the emergency department needs at least the same amount of personnel as a polytrauma (…), where without batting an eye, five to ten people are ordered.” [#3, male ED physician]

Furthermore, participants had experienced supervision of potentially aggressive patients as an effective measure, especially for individuals with substance misuse having limited impulse control. This could be carried out by students, patient transport service, or security staff, who could attend respective patients through all ED processes and respond to their requests (e.g., food, water, or visiting the washroom). Such one-on-one maintenance for psychiatric or demented patients was experienced to limit the use of physical restraints. Altogether, caring for aggressive patients was described to require personnel who were then not available to provide medical treatment.

Staff shortage was told to affect several other aspects of violence prevention besides staff rostering. For instance, more staff would be necessary in order to spare employees for training measures, reflect on past incidents, reduce the workload, or take regular breaks. All of this was described as necessary to reduce employees' stress level and thus equip them to better handle aggressive patients. Participants further stated that with more time per patient they would be able to recognise early warning signs for aggressive behaviour and apply preventive measures, since especially verbal de-escalation and catering to aggressive patients’ needs in a calm manner were described as time-consuming. Staff shortage in other divisions was described as well, which led to overcrowding and exit-blocks in EDs, e.g., due to waiting times for test results or because of delayed exit to other wards, hospitals, and treatment homes.

##### Security service

Security staff was called for support if employees needed help to deal with aggressive patients. The participants described advantages of security staff being permanently positioned in the ED. Firstly, security staff approached potentially aggressive patients early on and secondly, reduced the workload for ED personnel when they stayed with potentially aggressive patients in order to intercept all queries these patients might express. Thirdly, the mere presence of security staff was described as effective in reducing aggressive behaviour as well.

“Well, what is very nice is that now security service is sitting in front at the entrance area, because its presence alone sometimes helps a bit. You do not have to look yourself all the time because he is also looking (…). Or otherwise, if we have a psychiatric patient, who might be very restless because he absolutely wants to go outside to smoke et cetera, then we can also just resort to the security service, who will then accompany him. And then we are more relaxed because we know: okay he will be accompanied to go outside and they will also bring him back. That disburdens us a lot.” [#1, female ED nurse]

Security staff was further perceived as helpful if they were able to de-escalate verbally but could also intervene physically if necessary. However, some participants told there were restrictions that prohibited physical interventions from security staff and thus some patients were not daunted by them anymore. It was also mentioned that the presence of security staff could provoke aggressive patients even more, and uniforms might only suppress aggression that could erupt once security staff was gone. One participant had previously experienced security service in plain clothes as an effective measure and wished for its reintroduction. If security staff was available in the ED itself, they were mostly positioned in the entrance area, but in general on-call security service was more common. In that case, security staff was not perceived as helpful if they needed too long to reach the ED. The biggest constraints regarding security staff were if they had too little personnel to cover all their areas of application, or if their personality and physical appearance was not suitable for the job (e.g., inciting character, not trained for the job, not physically fit, or not willing to intervene). In EDs without security staff, participants wished for its implementation at least at night or on the weekends. Others wished to expand the time where security staff was available and wanted them to be present right by the ED instead of on-call duty. Overall, well organised security services comprising competent staff was perceived as very effective, while poorly organised security service that did not have enough capacities or unskilled personnel was perceived as unhelpful.

##### Police

Ultimately calling the police, in particular in cases of physical violence, was a common measure if neither employees nor security staff could control the aggressive patient. Calling the police in advance, in cases where violent behaviour was anticipated, was unusual. Direct dialling to the next police station was often possible and the police was described as an effective measure, since they arrived fast with the needed manpower, and were themselves experienced in dealing with aggressive people.

“As I said, if I have a 1.90m, muscular, young man who additionally has perhaps taken drugs somehow as a background, then I know: I need a bit more manpower and there I also need people who know exactly how to handle them. And even our psychiatric ward (…), they also call the police. So in my opinion that's the most effective or the most helpful means.” [#4, female ED nurse]

It was perceived as helpful if the police stayed throughout the treatment, but there were also participants stating that sometimes the police was not good in de-escalating and showed aggressive behaviour themselves. There were only few further demands regarding the police, e.g., if they generally took too long to arrive at the ED or did not stay to support aggressive patients’ treatment.

##### Guidelines and standard operating procedures (SOPs)

The majority of participants reported they had not come across a guideline or a standard operating procedure (SOP) regarding violence prevention in their ED. The existing guidelines and SOPs usually pertained to one specific aspect of violence prevention, e.g., how to take care of people with substance misuse or whom to inform in case of a violent incident. A comprehensive guideline was available in the quality management handbook of one ED and it included aspects from detecting early warning signs to dealing with already aggressive patients as well as documentation of the incident; however, it was not communicated and applied during daily work routines. The available guidelines and SOPs were usually not perceived as helpful due to insufficient descriptions of the correct demeanour, or because employees were not informed about them.

“There is a process instruction in the QM portal [quality management portal] (…). So it is just written there, yeah, to somehow ‘impinge on the situation in a de-escalating way’ – full stop. Whatever that is supposed to mean.” [#25, male ED physician]

Participants also stated that violent incidents were heterogeneous and that it would be difficult to write down a general guideline that would help with the correct behaviour in such diverse situations. Hence, it was also stated that basic principles of violence prevention were rather transferred verbally, along with reminding each other of the correct behaviour, instead of relying on guidelines and SOPs. However, other participants wished for a general guideline instead of having to muddle through the daily work routine without a concept regarding violence prevention.

“I think we still do too little. I think, it is a lot that you just accept and manoeuvre yourself through the daily routine. But a proper plan, we actually…, only few have.” [#19, female ED physician]

These participants wished for clear instructions regarding the right behaviour during violent incidents and demanded to be informed about the guidelines and SOPs relevant for their workplace on a regular basis, e.g., during induction period or training measures.

##### Screening tools

None of the participants were currently using a screening tool to predict aggressive behaviour of patients, and one participant stated that there were no good tools for early detection of potentially aggressive patients. Another participant described a related tool they had used in the past, namely a 10-point Likert scale to assess anxiety. Those patients showing higher anxiety levels had received more frequent information on the treatment process or had been assigned a higher urgency according to the triage system. The participant described that this had led to a decrease of violence, since anxiety was an important reason for aggressive behaviour. Similarly, other participants described they assigned a higher urgency according to their triage system to patients already showing aggression. However, one participant described the triage system currently used at their ED as unhelpful to predict aggressive behaviour, and another one addressed the need for a specific screening tool regarding violence in EDs.

“(…) there are no good scores or tools so far to identify these [aggressive] patients early on. And thus, there is of course no structured means to stop this escalation, because you do not recognise it at all.” [#24, male ED physician]

##### Structural conditions

There were two more superordinate organisational demands. Firstly, participants addressed the need for treatment centres specialised on individuals with substance misuse, since caring for the respective patients required personnel resources of the ED.

“(…) there I would wish for a reasonable way of dealing with intoxicated people and that this is done in a specialised manner and an institution established for this purpose. And namely with participation of the police, the judiciary, and also a somatic hospital.” [#3, male ED physician]

Secondly, there were many patients with minor medical complaints that insisted on treatment in EDs due to longer waiting times in outpatient clinics. Thus, participants described the benefits of integrating an outpatient practice into the hospital setting for referral of those patients.

“For four years now, we have the medical emergency service in our house, from the association of statutory health insurance physicians, in the emergency service practice. And a lot has already been de-escalated there. So since then, we also have considerable improvements because simply the waiting times are not so long.” [#20, male ED physician]

#### Individual-focused measures – employees

##### Training regarding violence prevention

Employee-focused measures mostly concerned trainings in violence prevention. The largest part of training measures – and also the part described as most effective – were communication techniques, like asking for the patients’ needs and validating their problems. Furthermore, recognising early warning signs for aggressive behaviour was a part as well in order to actively approach respective people and prevent escalations.

“Well, there are the early warning signs (…) that we train: restlessly walking up and down, hitting objects, fixed gaze, sweating, clenched fists. (…) The best de-escalation is the one that does not have to take place because he [the patient] does not escalate because instead you recognise him, and approach him, and dare to address him directly.”[#8, female ED nurse]

Other training measures aimed at understanding the patients’ and their attendants’ perspectives, which was further complemented with knowledge regarding diseases that increase the risk of aggressive behaviour (e.g., dementia, psychiatric diseases, or substance misuse). As a last resort, physical self-defence, the procedure of physical restraint, or recognising the right time to flee and alert others was also a part of training measures.

The available training measures were described to neglect employees’ own emotions in case of aggressive encounters, which also led to difficulties in following the suggested measures – especially under high pressure during a stressful workday. Thus, participants addressed the importance of teaching employees how to deal with emotions caused by infringing assertions of patients or attendants.

“(…) for this as well, we have too little training: how do I deal with it myself, if I was verbally attacked and hurt. It is always based on us not having any emotions at all and always just have to accept it. But it is not always like that, we are also only humans and sometimes just more susceptive for such, let’s say, verbal lapses of others than on different days.” [#17, female ED nurse]

Furthermore, it was described as important that the team was included in the process of developing and deciding about measures to implement, so that respective changes were more successful.

“The team always wants to be integrated and be part of the decision. I always find, performing efforts at persuasion and then introducing a proposal to the team, which the team works on and gets it off the ground that is actually the best to initiate change, volitional change.” [#8, female ED nurse]

**Fig. 1:**
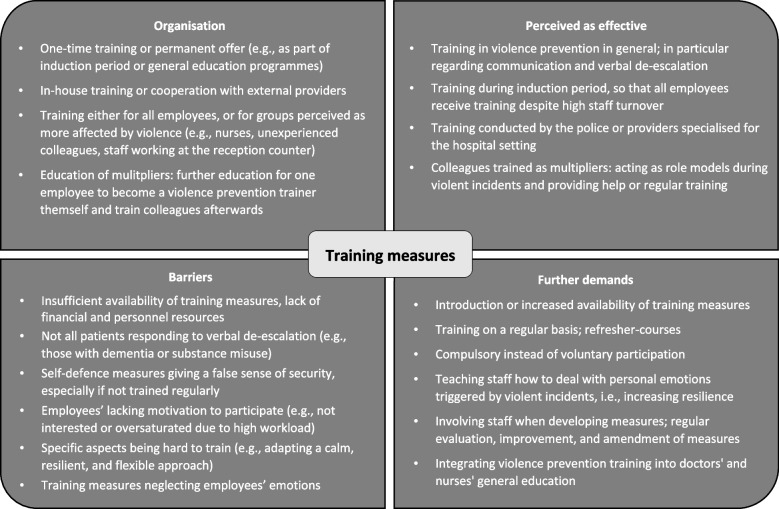
Employee-focused training measures for violence prevention

Further details regarding training in violence prevention are provided in Fig. 1.

Besides actual training measures, learning processes within the team were important, especially regarding the inner attitude and behaviour towards patients, which were described as hard to train but could be exemplified by experienced colleagues. Examples were knowledge transfer from experienced to new colleagues as well as supervision or debriefing sessions after violent incidents. The latter were also perceived as a possibility to learn from past incidents and reflect on the personal behaviour.

##### Staff care

The working climate was addressed as an important factor as well. This included support from supervisors in dealing with aggressive patients or taking care of employees’ health, well-being, and resilience in order to increase employees’ stress resistance.

“(…) it would not be my first priority to do training in the direction of de-escalation now, because you can do as much de-escalation training as you want, if you yourself are completely stressed, you will forget all of it, yes.” [#15, female ED nurse]

These aspects were perceived as crucial in order to equip employees to work in the ED until retirement age.

#### Individual-focused measures – patients and attendants

##### Communication

Participants described verbal de-escalation as an important and effective preventive measure, especially if it was applied as soon as early warning signs for aggressive behaviour were noticed in patients or their attendants.

“Conversations of course, that is the ultimate, that is what always helps in case of doubt, talking to people.” [#7, female ED nurse]

In general, the personal behaviour of staff was described to play an important role, meaning that it had a substantial influence on the course of a violent incident. Participants stressed the importance and effectiveness of approaching patients in a calm and friendly way without prejudices, if necessary, in a one-on-one situation and a quiet atmosphere. Even patients showing a more challenging behaviour like those with dementia or intoxication were described as easier to deal with if they were treated with time and patience to build up trust or were left alone for a while to adapt to the ED atmosphere. In general, two things were important when communicating with (aggressive) patients. Firstly, since longer waiting periods were a major reason for aggression, it was reported as helpful to make patients understand the reasons (e.g., patients are treated according to urgency, or employees from different disciplines may have different capacities). Secondly, participants felt it was effective to validate patients’ problems, show understanding, and ask for their individual needs to dissolve the confrontation, since eventually the patients visiting an ED were in an exceptional situation. Thus, to prevent aggressive behaviour, participants emphasised an increased information flow, e.g., transparency regarding ED processes and providing reasons for longer waiting periods. Along with measures for patient entertainment (e.g., free WLAN-access or TV), patient information was demanded in the form of video clips displayed in waiting areas, posters or sheets available in several languages, or patient information systems (e.g., showing the number of severely ill patients currently treated). Patients were also described to get aggressive if smaller requests were denied (e.g., food or going outside to smoke), so it was described as effective to accommodate these needs. However, due to time pressure in ED processes, participants told they were too stressed and had too little personnel resources to deal with patients in the manner described above.

“You usually don’t have the time for any long de-escalation talks, while in the background somehow three patients are almost dying. (…) and you are always running behind time.” [#10, male ED nurse]

Participants wished for more awareness in the general population regarding the amount of violent behaviour ED staff had to face on top of their stressful work, especially in terms of verbal violence. They asked to clearly state to patients and their attendants that ED staff had to be treated with more respect and that violent behaviour was not tolerable.

“So, what I think would be more useful from the start, what I still miss a bit, that this is reflected to the outside, that it becomes clearer to the population (…) that verbal violence is also not tolerated here. Because many people do not know that shouting and complaining at the highest level, that this is verbal violence and that we do not tolerate it, yes.” [#16, female ED physician]

Furthermore, participants wished for the general population to be educated about the fact that only medical emergencies should be treated at the ED and people with less pressing issues should visit practitioners from out-patient care to unburden EDs.

##### Access restrictions

Expelling aggressive patients and attendants from the ED was reported to be an effective way of dealing with violent behaviour. In addition, some participants described that official house bans were imposed as well, provided that the respective patient did not need immediate care. Access restrictions for attendants were implemented in all EDs, mostly due to the COVID-19 pandemic. The majority of participants wished these restrictions would be maintained, but with exceptions for patients who benefited from the presence of their attendants such as children.

“In my opinion, relatives in the emergency department, excuse my expression, do not belong there (…). Of course, it is nice for the patient, then he is not alone, but for the procedure and for the potential of aggression it is absolutely counterproductive in my view, because at the end of the day there is one more person who is constantly asking questions: (…) ‘why is nothing happening?’ (…). Which on the one hand disturbs the workflow, and on the other hand, of course (…), also promotes aggression. Of course, there are always exceptions: seriously ill patients, mentally retarded patients, very anxious patients. I think you have to make exceptions there, and we do that.” [#22, male ED physician]

##### Physical restraint

The last resort described by interviewees to deal with aggressive patients that needed medical treatment was restraint, i.e., physical restraint in order to prevent aggressive people from hurting themselves or others.

“What we always try to do first is to have a conversation. (…) In the one-on-one situation the psychiatrists try to reach a consensus. In many cases, this can already calm things down and it doesn't have to come to a situation of physical restraint or something. But the decision towards physical restraint (…) is then made by the psychiatrist.” [#24, male ED physician]

## Discussion

This qualitative study provides insights on available violence prevention measures in German EDs, their perceived effectiveness, barriers regarding their implementation, as well as further demands from the perspective of supervisors and employees working as nurses or doctors in German EDs. Measures commonly perceived as effective were related to communication or a well organised security service. While organisational measures mostly pertained to staff organisation, individual-focused measures primarily targeted employees’ behaviour when dealing with aggressive patients or aimed at informing patients as well as restricting access for attendants. Among technical measures alarm systems were common, while treating aggressive patients in a low stimulus and calming environment (e.g., single rooms) was highlighted regarding architectural measures. According to Weiland et al. [20] architectural changes in EDs are becoming more common, e.g., such specialised rooms for behavioural assessment. However, due to a limited body of research showing methodological weaknesses there is no sound body of evidence substantiating their efficacy [20]. In general, measures addressed in this study were not implemented based on scientific evidence, but due to previous incidents or because ED leaders had positive experiences with them. In this regard, Weiland et al. [20] described, that hospital managers have the urge to keep their staff safe and in view of lacking research they cannot wait for a sound body of evidence to implement a certain measure.

Moreover, a lot of interventions that participants described were not part of an officially implemented violence prevention guideline but could be rather described as based on common sense of participants. Those interventions were, for instance, explaining ED processes to impatient patients and their attendants, flight in case of physical encounter, or asking for help from experienced colleagues. Such personal strategies, which employees learn and implement on their own, have also been described in a previous study conducted by Charrier et al. [21], e.g., communication strategies aiming at a reciprocal understanding with the patient to keep the interaction congenial. These measures were commonly applied by participants of the current study as well and one reason that was addressed was that these strategies were not dependent on financial resources and could be implemented without organisational efforts. Furthermore, screening tools for violent behaviour were not applied in any of the EDs. Thus, recognising early warning signs was highly dependent on employees’ knowledge of human nature. Cabilan and Johnston [22] already provided an overview of available risk-assessment tools for violent behaviour. Among the five tools they discussed, they reported that four were rather aide-memoires or prompts to remind the staff of patient observation, but only one assessment tool (i.e., the Brøset Violence Checklist, BVC) predicted violence based on a score [22]. Two studies already provided results on the implementation of the BVC in EDs [23, 24]. In the study conducted by Patridge and Affleck [23], the assessment for risk of violence was conducted by an ED security officer. The positive predictive value, i.e., the probability of the patient committing a violent incident after being assigned a certain score, showed that over half of the patients assigned the highest cut-off score actually caused a violent incident afterwards [23]. Senz et al. [24] combined the implementation of the BVC with a management matrix suggesting de-escalation strategies like verbal de-escalation. The authors reported a significant reduction in unplanned violence-related security responses as well as an improved staff perception of organisational support [24].

The most commonly mentioned barriers with regard to the implementation of violence prevention measures were described to be a shortage of staff, a lack of financial resources, and a high workload. Previous research has already highlighted the high workload in EDs [25, 26]. On the one hand, ED employees are required to offer fast and proficient emergency care, while at the same time, they are expected to be sensitive when dealing with patients and their attendants [26]. On the other hand, the pressure of routine workflows and a lack of time can lead to scarce communication [26]. Likewise, interviewees of the current study described that the lack of personnel combined with the high patient inflow led to an increased workload and stress level. As a result, participants neither had enough time per patient nor the mental capacities to consider prevention measures like detecting potentially aggressive patients and calmly catering to their needs. Hence, another important aspect to consider for violence prevention became apparent, i.e., employee related factors. In their study, Schuffenhauer and Hettmannsperger-Lippolt [27] did not find a correlation between experienced violence and personality traits assessed with the Big Five Personality Test (B5T). Participants of the current study, however, described that personality traits were a decisive factor for violence prevention. For instance, employees could either be triggered by aggressive patients and promote escalations with their own behaviour, or they could be equipped with more resilience and the ability to reflect on their personal influence to prevent escalations. Likewise, Lindner et al. [5] summarised several staff related causes for violence in emergency care such as overexertion, lacking experience and communication skills, or the feeling of being personally attacked by patients and their attendants.

Participants of the current study described skills related to the inner attitude and personal behaviour as hard to learn or difficult to convey by means of training – instead, these were described to evolve with more work experience. Previous literature provides differing results on related training measures like those addressing behavioural (e.g., personal attitude) or educational (e.g., early warning signs for aggression) aspects. In their narrative review Heckemann et al. [28] reported that all studies found positive effects in at least one of three domains (individual attitude and confidence, incidence of aggression, as well as individual competence). However, changes regarding the incidence of aggression were not significant [28]. Similarly, an intervention review on education and training measures among healthcare workers showed that the evidence on their effectiveness is uncertain and sparse [29]: while education may improve personal knowledge and attitudes in the short term, the few studies available showed very uncertain evidence on the level of experienced aggression compared to no intervention [29]. Hence, more comprehensive approaches are needed, e.g., based on a combination of environmental, organisational, and individual-focused interventions. Such multi-dimensional approaches were already proposed by previous authors [10, 30], and suggested by official guidelines [9].

### Implications for practice

In the current study, a lack of comprehensive violence prevention guidelines became apparent. Guidelines or SOPs were, for instance, unavailable, unknown, or perceived as unhelpful in the current form. Therefore, guidelines tailored to the needs of the respective ED need to be developed and made available to all employees. More importantly, these guidelines should be communicated and practiced on a regular basis so that ED staff would feel prepared for violent incidents. Although there were accounts of violent incidents being very heterogeneous, such guidelines would provide a general course of action as a common ground for all employees on how to prevent and handle violent incidents. An essential part of such guidelines and SOPs could be the implementation of screening tools to specifically target patients identified as potentially aggressive. In this way, the focus would shift from dealing with violent incidents to preventing them.

Moreover, since the most mentioned demand was more staff, there is a need to disengage from economic efficiency in EDs when it comes to personnel resources. The participants did not make excessive claims, but for instance stated that employing one additional nurse per shift would help to take account of violence prevention. Recommendations for staffing of nurses in EDs were recently published by an alliance of several emergency care societies from Germany, Austria, and Switzerland [31], which could be applied as a reference value. Moreover, employees need to be involved when establishing preventive measures, to increase the motivation to participate. Furthermore, clear responsibilities should be established to promote violence prevention, e.g., ED-leaders having various other obligations can transfer respective tasks to a responsible staff member. The matter of violence prevention should further be embedded in daily routines, e.g., past incidents could be discussed during staff meetings in order to learn from them.

There are further implications regarding the general structure of the healthcare system to combat high workloads due to patient influx. In general, concepts to target overcrowding and exit-blocks need to be implemented, since previous research identified overcrowding as a main risk factor for violence in EDs [32]. This requires cooperations with different hospital wards, psychiatric institutions, and nursing homes in order to transfer patients on time. In addition, the implementation of outpatient practices close to the ED is necessary to take over less ill patients. Likewise, the establishment of specialised institutions to take over individuals with substance misuse is recommended, in order to reduce the potential for aggressive behaviour as well as the workload for ED staff.

### Implications for further research

In order to adapt violence prevention measures to the structural conditions of ED settings, differences in experiences with prevention measures between those settings need to be studied, e.g., based on the emergency care levels according to the G-BA [16]. Such structural differences became apparent in the current study, since participants reported to experience fewer violent incidents if the ED was smaller or located in a more rural area as, for instance, potentially violent patients were already known.

Three previous literature reviews on interventions for violence prevention in EDs found that the body of available literature is limited and thus, the evidence is sparse [10, 11, 33]. The current study can contribute valuable insights on the perception of respective measures. However, the lack of knowledge on their effectiveness puts clinicians in a difficult position when implementing violence prevention measures [33]. Nevertheless, there is a high demand for future research to monitor and evaluate the implementation of various violence prevention measures in different ED settings in order to provide a solid foundation for recommendations. Further research is also needed to refine and develop screening tools that can be integrated into ED routines, due to weaknesses of currently available tools: Cabilan and Johnston [22] reported that the BVC does not recognise important factors such as patients’ history or clinical presentation and in addition, none of the tools they found were designed to predict verbal violence.

### Strengths and limitations

To the authors’ best knowledge, this is the first qualitative study analysing violence prevention measures in German EDs. The qualitative approach allowed for profound insights into perceived benefits and detriments of available measures and revealed opportunities to improve violence prevention in EDs. Furthermore, this study considered perspectives from different professions (i.e., doctors and nurses) and staff members with and without leadership positions in EDs.

Several limitations need to be considered when interpreting the results of the current study. Due to the qualitative study design, the findings are not generalizable, as the results are based on the subjective perspectives of a limited sample. In addition, transferring the results to populations from countries with different emergency care systems might not be plausible. More bias might be present in the sample, since participants from EDs with a comprehensive care level were overrepresented, while EDs run by commercial sponsors were underrepresented.

Furthermore, the study was conducted during the COVID-19 pandemic which might have influenced the level of aggression occurring in EDs, e.g., when it comes to asserting access restrictions. However, from the interviews as well as from a study conducted by Schablon et al. [12], it seems that nearly equal proportions of participants experienced an increase as well as a decrease of violence and aggression, while others felt the level of aggression did not change during the pandemic. Thus, the influence of the pandemic on the results should be minimal. In addition, there might have been a self-selection bias, due to which only those might have signed up for the interviews who are most affected and thus interested in the topic, therefore giving the impression of German EDs being insufficiently prepared for violent incidents. On the other hand, participants were also contacted through their supervisors or were supervisors themselves. Thus, bias might have been introduced to the sample if supervisors had tried to impart a good impression of their efforts regarding violence prevention, ultimately leading to a better image of preparedness for violence in German EDs than there actually is. However, it seemed that all participants were genuinely interested in pointing out current weaknesses in order to improve violence prevention measures in German EDs.

## Conclusions

Although in most EDs certain violence prevention measures were applied, comprehensive guidelines were lacking and screenings for violent behaviour were not performed. Besides guidelines tailored to the specific requirements of the ED, clear responsibilities need to be established, and violence prevention needs to be embedded in daily work routines. Furthermore, the workload needs to be decreased in order to facilitate violence prevention, either by reducing the patient inflow or by employing more doctors and nurses. Although sound scientific evidence on the effectiveness of prevention measures is still lacking, action needs to be taken in order to decrease high prevalence rates of violence in EDs. Prevention guidelines should combine environmental, organisational, and individual-focused measures to sustainably increase employees’ physical and mental health as well as their professional quality of life.

## Data Availability

The datasets generated and analysed during the current study are not publicly available due to German national data protection regulations. They are available from the corresponding author on reasonable request as well as with permission of the funding organisation and their supervisory authorities.

## Abbreviations

B5T: Big Five Personality Test
BVC: Brøset Violence Checklist
CI: Confidence interval
COREQ: Consolidated Criteria for Reporting Qualitative Research
DGINA: Deutsche Gesellschaft für Interdisziplinäre Notfall- und Akutmedizin, German Society for Interdisciplinary Emergency and Acute Medicine
ED: Emergency Department
G-BA: Gemeinsamer Bundesausschuss, Federal Joint Committee
ILO: International Labour Organization
SOP: Standard Operating Procedure
